# Mannitol mediates the mummification behavior of *Thitarodes xiaojinensis* larvae infected with *Ophiocordyceps sinensis*

**DOI:** 10.3389/fmicb.2024.1411645

**Published:** 2024-08-19

**Authors:** Wenmin Chai, Xianbing Mao, Chunfeng Li, Liancai Zhu, Zongyi He, Bochu Wang

**Affiliations:** ^1^Key Laboratory of Biorheological Science and Technology, Ministry of Education, College of Bioengineering, Chongqing University, Chongqing, China; ^2^Chongqing Xinstant Biotechnology Co., Ltd., Chongqing, China; ^3^State Key Laboratory of Silkworm Genome Biology, Southwest University, Chongqing, China

**Keywords:** *Thitarodes xiaojinensis*, *Ophiocordyceps sinensis*, mummification, metabolic components, mannitol

## Abstract

**Introduction:**

Parasites can facilitate their own spread and reproduction by manipulating insect hosts behavior, as seen in the interaction between *Thitarodes xiaojinensis* and *Ophiocordyceps sinensis*. Infection by *O. sinensis* leads to the mummification of *T. xiaojinensis* larvae, but the underlying mechanisms remain mysterious.

**Methods:**

The morphology of *O. sinensis* infected larvae and fungal growth were first observed. Subsequently, the metabolite changes in the larvae before and after infection with the fungus were analyzed by LC/MS and targeted metabolomics. The expression of mannitol-related genes was detected using RT-qPCR, and morphological changes in larvae were observed after injection of different concentrations of mannitol into the *O. sinensis*-infected larvae.

**Results:**

Significant changes were found in phenotype, fungal morphology in hemocoel, larval hardness, and mannitol metabolites in infected, mummified 0 h larvae and larvae 5 days after mummification behavior. Surprisingly, the occurrence of mummification behavior was accompanied by fungal dimorphism, as well as the absence of mannitol in both infected and non-infected larvae, until the initial accumulation of mannitol and the expression of mannitol-associated genes occurred at the time of mummification behavior. The presence of mannitol may promote fungal dimorphism to mediate changes in fungal toxicity or resistance, leading to the end of the fungus-insect coexistence period and the incidence of mummification behavior. Furthermore, mannitol injections increase the mummification rate of the infected larvae without significant difference from the normal mummification phenotype.

**Discussion:**

This finding suggests the importance of mannitol in the mummification of host larvae infected with *O. sinensis*.

## Introduction

Chinese cordyceps, a rare fungus known as the “insect within the grass, and the grass within the insect,” holds a prominent position in the history of Chinese medicine. The distinctive feature of Chinese cordyceps is its complex biological properties, which not only contains the structure of an insect, but also incorporates the vitality of a fungus. This Chinese herb is highly regarded for its multiple pharmacological properties including anticancer, antioxidant, antibacterial and immunomodulation (Yi et al., 2015). The formation process of Chinese cordyceps is a marvelous parasitic phenomenon in nature, in which a lepidopteran worm from the genera of *Hepialus* and *Thitarodes* was infected by *Ophiocordyceps sinensis* (*O. sinensis*), and forms a mycelium in the body of the host (Cheng et al., 2016; Wu et al., 2020). As the mycelium matures, it manipulates the behavior of the host larvae, causing them to migrate toward the soil surface and form fruiting body on the larval head. This process eventually leads to the death of the larvae, and the fungus takes this opportunity to proliferate, forming a unique host cadaver mummy containing fruit body of entomopathogenic fungus that we know as Chinese cordyceps (Wang and Yao, 2011). Although the medicinal value and formation mechanism of Chinese cordyceps have been extensively studied, the molecular mechanisms that lead to changes in insect behavior during this fungus-insect interaction have not been fully revealed.

The fact that Chinese cordyceps is only found in the Tibetan Plateau at altitudes of 3,000 to 5,000 meters indicates that both ghost moth larvae and *O. sinensis* are psychrophilic (Xia et al., 2015). The price of Chinese cordyceps, which is about 60,000 USD per kilogram, is exceptionally costly due to its scarcity and medical value (Lei et al., 2015). Though limited progress has been achieved due to the mystery surrounding life cycle of Chinese cordyceps and the challenge of creating a laboratory model of an entomopathogenic fungus and its host, artificial cultivation holds the potential to promote the production of Chinese cordyceps (Qin et al., 2018). Previous studies have focused on the isolation and genetic identification of various *O. sinensis* strains (Xiang et al., 2014; Xin et al., 2018; Li et al., 2019), the extraction and pharmacological mechanisms of active ingredients (Chen et al., 2014; Zhao et al., 2014; Liu et al., 2016), and large-scale breeding of *O. sinensis* and ghost moth larvae (Meng et al., 2017; Ríos-Díez et al., 2017; Ruo-Yao et al., 2018; Zhu et al., 2019). These studies have usually studied the fungus and the host independently, and little has been done to understand the mechanisms underlying the larval mummification behavior (Shrestha et al., 2010). Therefore, solving the riddle of the mummification behavior of Chinese cordyceps will contribute to artificial cultivation's yield (Li et al., 2018).

In nature, fungal invasion usually results in altered host metabolism, and we hypothesized that certain components were involved in the mummification behavior of *T. xiaojinensis* (Xia et al., 2023). Carbohydrates play a crucial role in the host-parasite system, where they not only underlie the energy supply of host cells, but are also involved in immune regulation and the control of inflammatory responses (Verissimo et al., 2019). Parasites often rely on host carbohydrates as a source of nutrients, and at the same time may manipulate host metabolic pathways to optimize the environment for their own survival, or even alter host behavior to facilitate their spread (Campetella et al., 2020). For example, the xylose isomerase of *Lactobacillus brevis* recapitulates the motility effect of microbial colonization by regulating sugar metabolism in Drosophila melanogaster (Chng et al., 2017). In this study, we used mass spectrometry to compare the differences in metabolic components of ghost moth before and after the mummification, and found that mannitol is an important metabolite. Mannitol, a bioactive component in Chinese cordyceps, plays an essential role in the physiological activities of its species perpetuation process (Lo et al., 2013). Further experiments involving the direct injection of a 4 mM concentration of mannitol into a ghost moth's hemocoel confirmed the ability of mannitol in mediating the fungal infection of the larvae leading to larval mummification. This result suggests that mannitol is not only involved in the growth and development of the fungus, but may also play a crucial role in the manipulation of host behavior by the fungus.

The aim of this study is to explore the role and importance of mannitol in mediating the manipulation of larval mummification by the fungus *O. sinensis*, with a view to providing new insights into the understanding of this complex biological interaction.

## Materials and methods

### Experimental insects and fungi

The larvae of ghost moths, *T. xiaojinensis* was originally collected from alpine meadows (Xiaojin County, Sichuan Province, China), and then reared in the laboratory at 15°C with 80% relative humidity for more than two generations (Wu et al., 2020). Larvae of the third generation were collected for later research.

*O. sinensis* was isolated from fresh Chinese cordyceps collected from alpine meadows (Xiaojin County, Sichuan Province, China). The *O. sinensis* fungi were cultured in dextrose peptone medium at 18°C. Fifth instar larvae (head capsule 2–2.5 mm, body length 0.28–3.3 cm, weight 0.20–0.26 g) were pricked with a glass capillary tube containing 2 μL diluted fungal suspension (3 × 10^6^ blastospores/μL), and then transferred to plastic cups and feed individually in a rearing chamber at 15°C with 80% relative humidity.

### Hardness measurement

The hardness of larvae was measured using a digital display shore hardness tester (LXD-C, Deqing SHENGTAIXIN Electronic Technology Co., LTD) with a shore scale of 0–100 HC (*N* = 8 for each group). Briefly, the pressure needle of the hardness tester was smoothly inserted into the first, third and fifth air holes corresponding to the pedipalps, and the value was taken after the pressure foot was in complete contact with the sample for 1 second. Eight worms were taken at each time point and the statistics were averaged. Day 0 is the moment of mummification behavior, with the larva's body stopping moving and only their mouthparts remaining active.

### Observation of *O. sinensis* developmental stages

The inoculated *T. xiaojinensis* larvae of 6^th^ and 8^th^ and at 0, 1, 3, and 5 days post mummification (*N* = 8 for each group) were selected for observation. We extracted 20 μL hemolymph from each larva and diluted it into 1 mL of 0.1% Tween 80 to obtain the hemolymph diluted solution. After adding the 20 μL hemolymph dilution to the glass slide, the morphology of the organisms was observed under a microscopy (Olympus X71) at 10 × 40 magnification. The percentage was calculated in triplicate for each larva.

### LC-MS analysis on metabolism difference

Eighth instar infected and uninfected larvae before mummification, infected larvae at the moment of mummification on Day 0, and mummified cadavers begin to harden after the mummification behavior on Day 5 were selected for metabolism difference analysis (*N* = 8 for each group). In brief, larvae were lyophilized to constant weight and then crushed into a fine powder. Hundred mg of sample was extracted with 0.6 mL of 80 % methyl alcohol (Aladdin, HPLC grade), vortexed for 1 min and twice grinding at 50 Hz. After 15 min of sonication, the product was centrifuged at 12,000 rpm for 10 min at 4°C to obtain the supernatant. Finally, 200 μL of the supernatant was determined on a Thermo-Fisher UPLC system (Thermo-Fisher, U3000) coupled with a mass spectrometer (Thermo-Fisher, QE Plus).

### Targeted metabolomics

Five stages of larvae, including infected larvae at 6th and 8th instar, and Day 0, 1 and 5 mummified cadavers (*N* = 8 for each group), were used to targeted metabolomics for saccharides and alcohols. The samples were lyophilized as previously described, ground into a powder, and then 100 mg of powder was dissolved in 4 mL of deionized water. The samples were then sonicated for 30 min, and centrifuged at 16,000 rpm for 5 min at 4°C to obtain the supernatant. After filtering through a 0.22 μm microporous membrane, 50 μm of the filtrate was dissolved in 950 μm of deionized water to prepare the injection sample. In addition, glucosamine, trehalose, glucose, and mannitol (Aladdin, HPLC grade) were dissolved in deionized water at a concentration of 0.1 mg/mL and used as standard controls. HPLC analysis was conducted on a Thermo-Fisher UPLC system (Thermo-Fisher, U3000) using a reversed-phase column [xtimate^®^ sugar-H, 7.8 × 300 nm, 5 μm, S/N: 5U1701503 (HPLC-15-005^#^)]. The parameters were as follows: 10 μL for the injection volume; 80°C for the column temperature; 0.3 mL/min for the flow rate; and 10 Hz CAD monitoring of the eluates. Deionized water was used as the mobile phase.

### Real-time quantitative polymerase chain reaction

Total RNA of larvae was extracted using TRIzol (Invitrogen) method, and the RNA concentration was determined using a NanoDrop-2000 spectrophotometer (Thermo Scientific). RNA was then reverse transcribed into cDNA using Transcriptor cDNA Synth. kit 2 (Roche, Basel, Switzerland) according to the manufacturer's instruction. Real-time quantitative PCR was performed using primers specific for Fructose kinase (FK), Phosphoglucose isomerase (GPI), Hexokinase (HK), Mannitol-1-phosphate dehydrogenase (MPD), and GAPDH (Sangon, Shanghai, China), based on the FS Essential DNA Green Master (Roche). Primer sequences were shown in Supplementary Table S1. Reactions were performed in triplicate. Finally, the results were analyzed according to the 2^−ΔΔCT^ method and normalized by GAPDH.

### Treatment of larvae with mannitol injection

Three different stages of larvae were taken and used for this experiment, including 8th instar larvae, larvae with capsule-shaped blastospore and larvae with pod-shaped blastospore (*N* = 100 for each group) on hemolymph microscopy. Mannitol with concentration of 20%, 15%, 10%, and 5% were prepared as the experimental group. Erythritol with concentration of 20%, sorbitol with concentration of 20%, and sterile water were used as control groups. The 20 μL sample solution was injected into the larvae body through stomata using a customized microinjection system (Zhonglou, model 0.33 mm, Instrument Factory of West China Medical University). The concentrations used were corresponding to 4 mM, 3 mM, 2 mM, and 1 mM, respectively. The percentage of mummification and pseudomycelia formation of the larvae (*N* = 8) was then calculated at 24 h, 72 h, 120 h, and 240 h post injection, respectively. Finally, the morphology and hardness change of the larvae before and after the mummification behavior, as well as the developmental stages of *O. sinensis* in hemocoel were observed.

### Statistical analysis

Statistical data were analyzed using SPSS 22.0 statistical software (IBM Corporation, USA), and GraphPad Prism 9.0 software (Dotmatics, USA) for graph generation. All experiments were repeated three times independently. All data was expressed as mean ± standard deviation (SD) and analyzed by Student's *t*-tests. *P* < 0.05 was defined as statistical significance.

## Results

### Morphology and hardness change of *T. xiaojinensis* larvae before and after the mummification behavior

**Figure 1:**
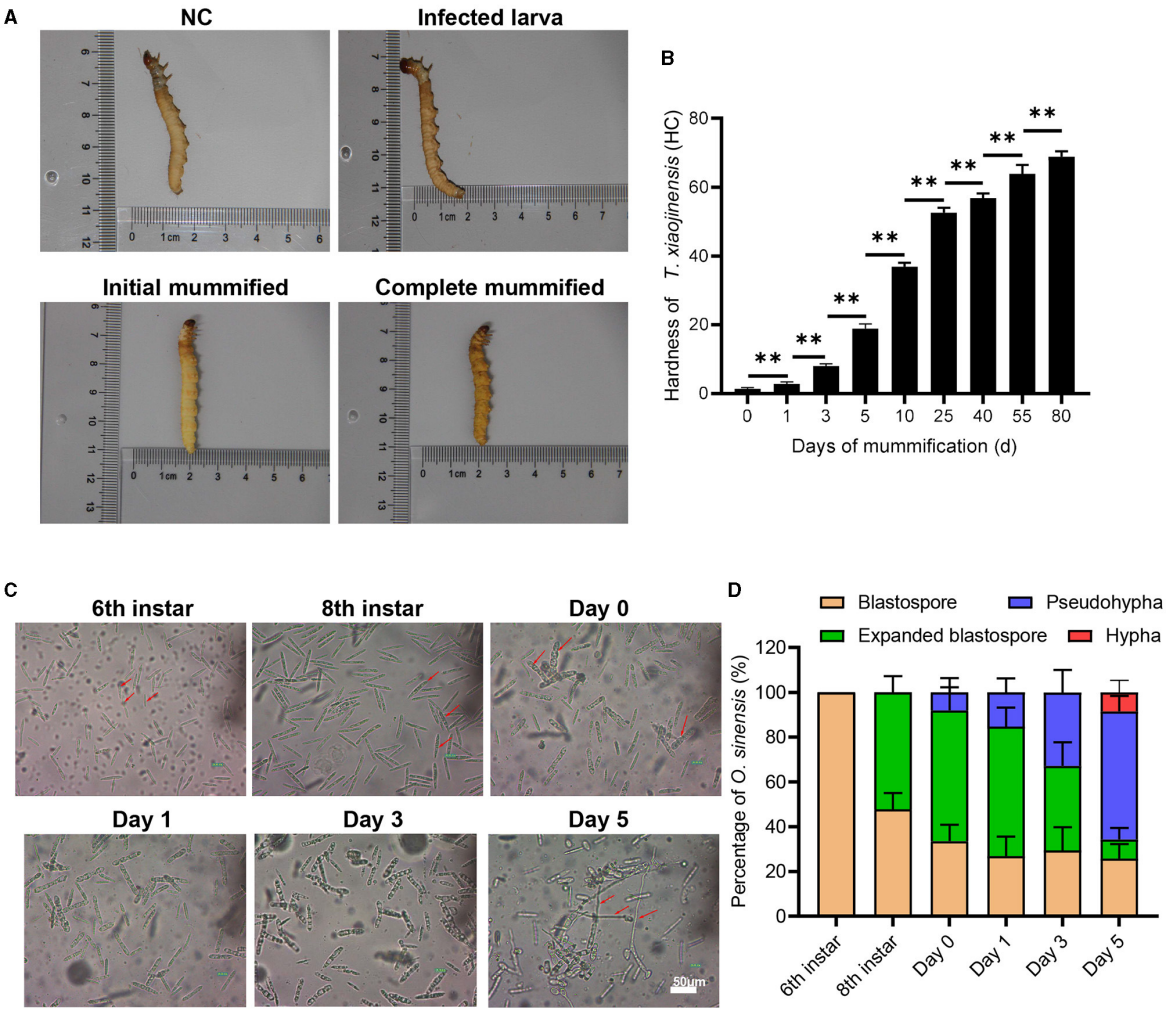
The morphology of *T. xiaojinensis* larvae and *O. sinensis* before and after mummification behavior. **(A)** The morphology of larvae at different stage. **(B)** The hardness of larvaes. **(C)** The morphology of *O. sinensis*. **(D)** Percentage of *O. sinensis* in the hemocoel of *T. xiaojinensis*. Compared with previous stage, data are mean ± SEM. **p < 0.01, *N* = 8. Representative morphological pictures are shown.

We first observed the morphology and characteristics of eight individuals per group of *T. xiaojinensis* larvae before and after mummification behavior, including uninfected 8th instar, infected 8th instar (before the mummification behavior), infected larvae at the moment of mummification behavior (the 8th instar larvae entered the agonal stage, defined as Day 0) and mummified cadavers after the mummification behavior (about 5 days post larvae death, defined as Day 5). As shown in Figure 1A, no obvious difference in the appearance or movement was found between the uninfected and infected 8th larvae. When growing to about 25 days, the larvae begin to crawl slowly, feeding stops and they reach to 1–3 cm away from the soil surface with their heads facing upwards, the worm mummified, followed by stopping of movement of the head and mouthparts until complete death. Initial mummification is completed 5 days after death, and as time goes on, it mummifies completely, eventually forming Chinese cordyceps about 80 days. Subsequently, we measured the hardness of the test insects. The hardness values of infected larvae and uninfected larvae were approximately 1.4 HC, and gradually increased after the mummification behavior: at the early stage, ~2.9 HC on day 1 increased rapidly to ~8.0 HC on day 3, and at the middle stage, ~18.9 HC on day 5 to ~56.8 HC at day 40, increased continuously. After that, the rate of hardness increases gradually slowed down (Figure 1B). With a hardness value of ~68.9 HC, the Chinese cordyceps produced at day 80 in the latter stage. These results arouse our interest in exploring the causes leading to the mummification behavior in the larvae infected by *O. sinensis*.

### Development of *O. sinensis* in the hemocoel of *T. xiaojinensis* before and after the mummification behavior

Using eight individuals per group, we also observed the morphology of *O. sinensis* involved in mummification behavior happened. The development of *O. sinensis* was divided into three stages based on morphology: blastopore, pseudohypha, and hypha. At the 6th instar of larvae, only primitive-type blastospores with dark fusiform shape were visible in hemocoel (Figure 1C). The number of blastospore gradually accumulated after 2–3 months of multiplication to the 8th instar, half of which matured into enlarged blastospore (Figure 1D). Subsequently, when larvae entered agonal stage on day 0 of mummification behavior, a few blastospores developed into swollen pol-like multinuclear segmented filaments (8.1%), and the proportion of pod-like pseudohypha in the hemocoel increased rapidly from 15.3% on day 1 to 32.8% on day 3. Finally, 8.5% of the pseudohyphae sprouted into elongated hypha when the larvae were thoroughly mummified at day 5 after mummification behavior. Therefore, the mummification behavior of infected larvae occurs accompanied by the development of *O. sinensis* in the larvae's hemocoel.

### Metabolism analysis of infected *T. xiaojinensis* before and after the mummification behavior

**Figure 2:**
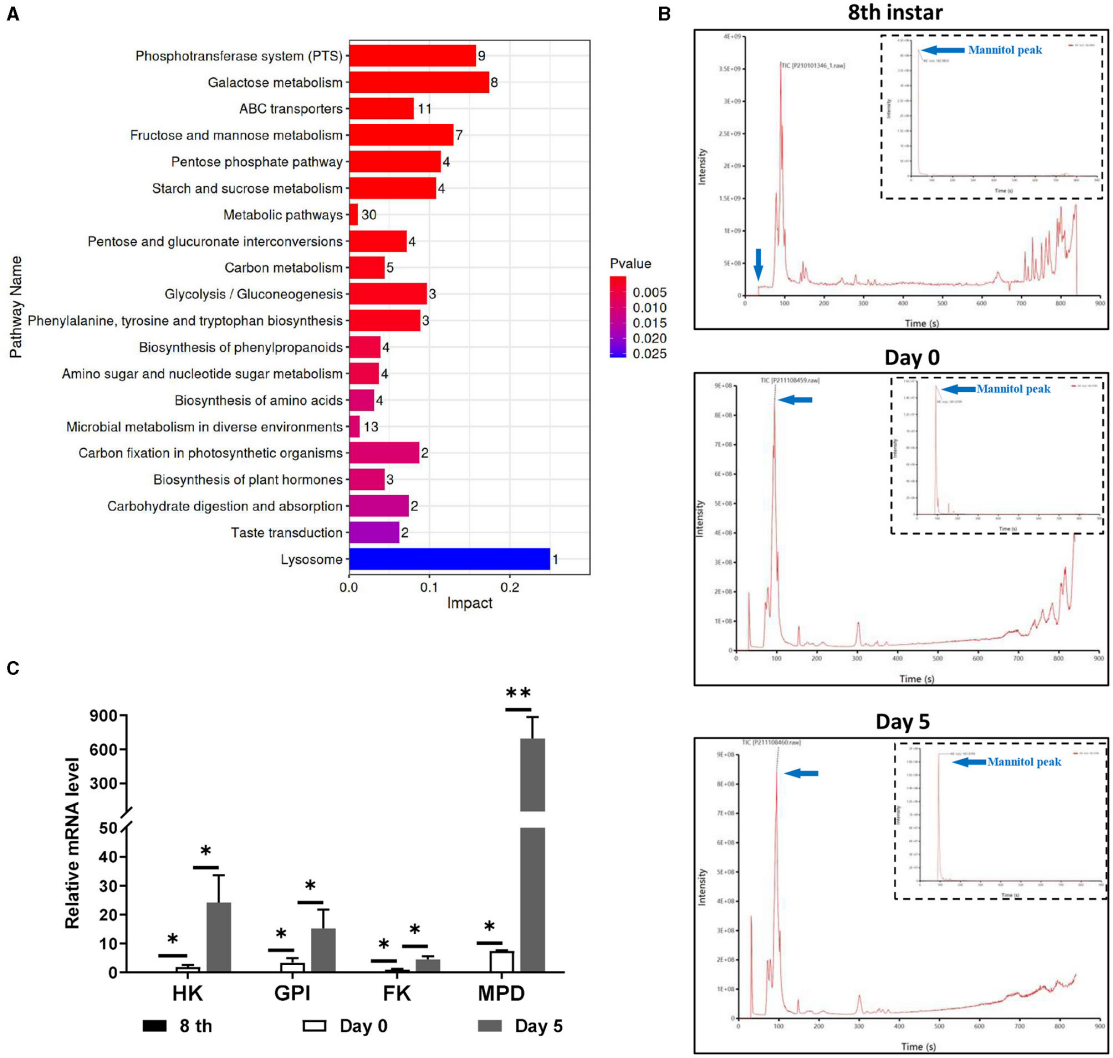
Metabolism difference analysis of infected *T. xiaojinensis* before and after mummification behavior. **(A)** KEGG analysis. **(B)** Mass spectrometric pattern at 8th instar, day 0 and day 5 after mummification behavior. **(C)** Expression of mannitol synthesis related gene. Compared with previous stage, data are mean ± SEM. *p < 0.05, **p < 0.01, N = 8.

There were metabolic changes that matched to the growth and development of *O. sinensis* in the hemocoel of infected *T. xiaojinensis*. We then collected morphologically distinct larvae on 8th instar infected and uninfected larvae, days 0 and 5 before and after the mummification behavior for untargeted metabolomics, with 8 individuals per group. Glucosamine, glucose, trehalose, mannitol, and xylitol were among the saccharides and alcohols that displayed notable variations in content between day 0 and 5, according to bioinformatic analysis (Supplementary Figure S1). KEGG analysis further revealed that these differential metabolites were mainly enriched in several signaling pathways related to carbohydrate metabolism and biosynthesis (Figure 2A). Interestingly, we discovered that mannitol and glucose were active in the metabolic network (Supplementary Figure S2), suggesting that mannitol may play important roles during mummification of *T. xiaojinensis*.

From the 6th instar to day 5 post mummification, targeted metabolomic experiments were performed constantly to monitor the metabolic properties of mannitol in greater detail. The results showed that mannitol was first identified on day 1 post mummification, followed by a rapid increase on day 5 post mummification (Figure 2B). The characteristic peak of mannitol, which was absent at 8th instar, first emerged on day 0 post mummification and expanded on day 5 post mummification (Table 1). RT-qPCR was performed to detect the expression of genes related to mannitol synthesis, and the results showed that FK, GPI, HK and MPD were not expressed in the 8th instar larvae (Figure 2C). With the appearance of pseudohyphae and mannitol, low levels of FK, GPI, HK, and MPD could be detected on day 0 at the moment of mummification behavior, and the levels of these markers increased dramatically when growth continued to day 5. These results demonstrate that mannitol appears only after the mummification behavior occurring of *T. xiaojinensis*, and that its expression gradually increases during the subsequent formation process.

**Table 1:** Targeted metabolomics analysis results of saccharides and alcohols (*N* = 8).

**Stages**	**Glucosamine (%)**	**Trehalose (%)**	**Glucose (%)**	**Mannitol (%)**
6th	12.43 ± 0.027	1.04 ± 0.005	1.21 ± 0.006	—
8th	10.46 ± 0.011	1.86 ± 0.004	1.20 ± 0.006	—
Day 0	14.20 ± 0.021	2.09 ± 0.004	1.28 ± 0.008	—
Day 1	11.63 ± 0.027	0.58 ± 0.003	0.82 ± 0.003	0.43 ± 0.000
Day 5	13.45 ± 0.032	—	—	1.44 ± 0.002

The data is presented as mean ± SD. — represents that no samples were detected for this indicator.

### Effect of mannitol injection on mummification behavior of infected *T. xiaojinensis*

**Figure 3:**
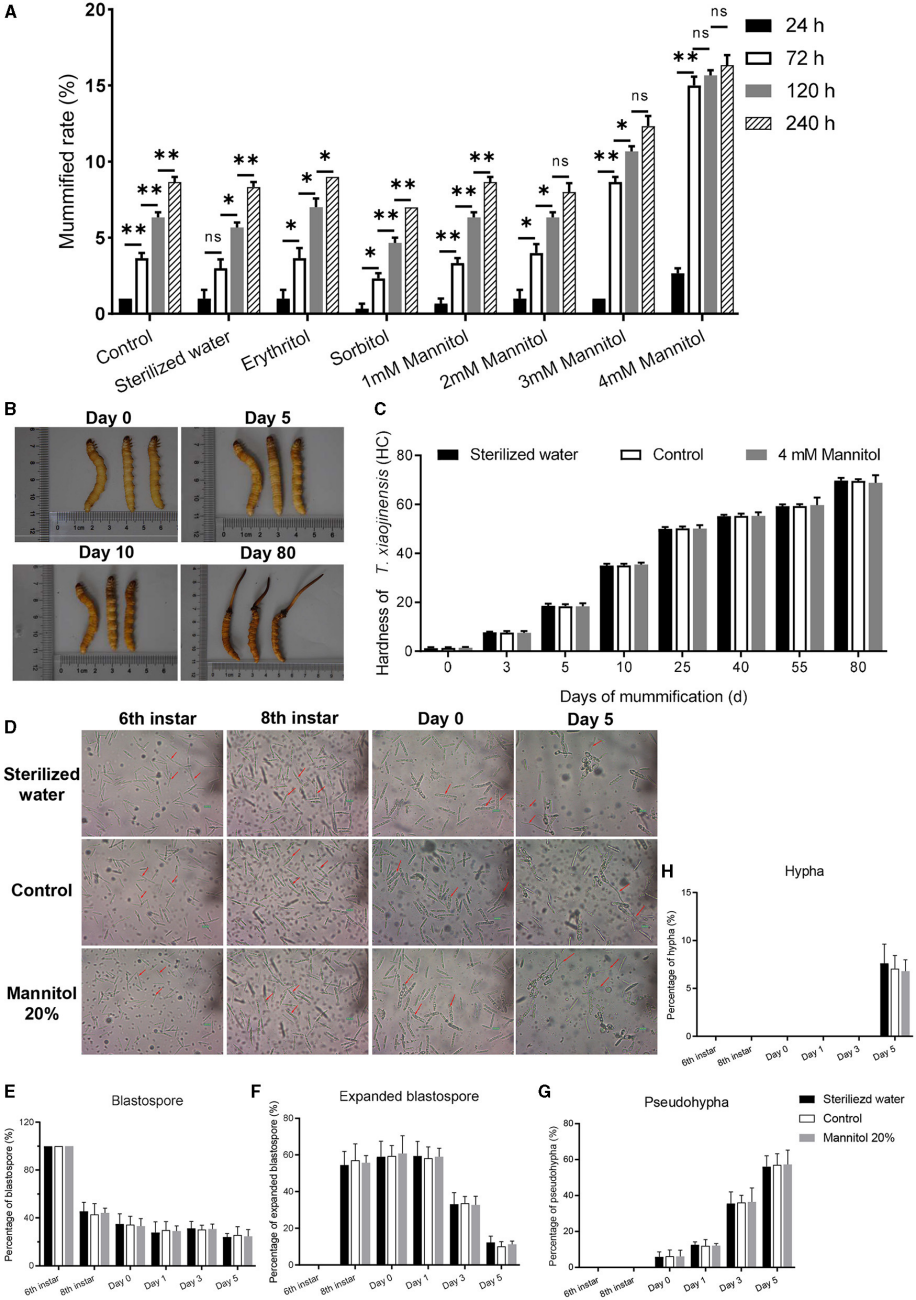
Effects of mannitol injection before and after mummification behavior of infected *T. xiaojinensis* with *O. sinensis*. **(A)** Mummification rate of different treatment and time post injection. **(B, C)** The morphology and hardness measurement of *T. xiaojinensis* larvae post injection of 4 mM mannitol. **(B)** Left: sterile water treatment, center: uninjected, right: mannitol treatment. **(D)** Development of *O. sinensis* in the hemocoel of *T. xiaojinensis* before and after mummification behavior post injection of 4 mM mannitol. **(E–H)** Proportion of blastospore **(E)**, expanded blastospore **(F)**, pseudohypha **(G)** and hypha **(H)** in different stages under different treatment conditions Data are mean ± SEM. *p < 0.05, **p < 0.01, *N* = 8.

To further clarify the effect of mannitol in the mummification behavior of *T. xiaojinensis* larvae, we injected 20 μL different concentrations of mannitol into the hemocoel of 8th instar larvae infected with *O. sinensis* (*N* = 100 for each group). When 4 mM mannitol was injected into 8th instar larvae, the proportion of the mummification at 24–240 h was much higher than the same volume of other concentrations of mannitol or analog injection (Figure 3A). The mummification rate reached peaked at 72 h after injection of 4 mM mannitol and then increased slowly. Moreover, injection of the same volume 3 mM mannitol also accelerated the rate of larval mummification occurring, but slower than 4 mM mannitol treatment. In contrast to the control group, there was no difference in the rates of mummification for low concentrations of mannitol (1 mM and 2 mM), mannitol analogs, and sterile water injection.

The hardness value of *T. xiaojinensis* larvae injected with 4 mM mannitol increased dramatically during the early to middle period, rising from 1.4 HC on day 0 to 16.3 HC on day 5, and continued to increase to 48.6 HC on day 40. And then slowly increased to 68.6 HC at day 80 (Figures 3B, C). Infected larvae with constant morphology, age, and size were injected with mannitol, which increased the rate of mummification and accelerated the occurrence of mummification behavior. However, the overall morphology of the injected larvae remained the same as that of control and sterile water-injected larvae.

More than half of expanded blastospores were visible when the larvae grew to 8th instar (Figures 3D–F). When the larvae entered agonal stage at day 0 of the moment of mummification behavior, 6.1% of the blastospores grew into pseudohypha, which increased rapidly to 36.4% on day 3 (Figure 3G). Finally, 6.8% of the pseudohypha germinated into elongated hyphae at day 5 of complete mummification (Figure 3H). Subsequent analysis revealed no significant difference for low concentration mannitol injection, sterile water injection and control treatment on the development of *O. sinensis*. In summary, mannitol treatment accelerated the mummification behavior of *T. xiaojinensis* larvae occurs.

Compared to the control group, the mummification rate was significantly higher after injection of 4 mM mannitol, but only about 15% of the mummification rate was achieved at 72 h after injection, and the other 85% remained without rapid mummification. Furthermore, we shortened the observation period by performing hemocoel microscopy of the infected 8th instar larvae injected with mannitol every 5 days until the onset of mummification behavior. Based on the observed results, the blastospores were broadly categorized as fusiform blastospore, enlarged blastospore, capsule-shaped blastospores, and pod-like blastospores. Moreover, the fusiform blastospore in the hemocoel of infected 6th instar larvae develops into enlarged blastospore, capsule-shaped blastospores and pod-like blastospores in the 8th instar, with consequent pseudohypha formation and mummification behavior occurring (Supplementary Figure S3). The reason may be that the different developmental stages of fusiform blastospore produce different mummification factors to induce the onset of mummification behavior.

### Validation of mannitol injection improving the mummification rate of infected *T. xiaojinensis*

**Figure 4:**
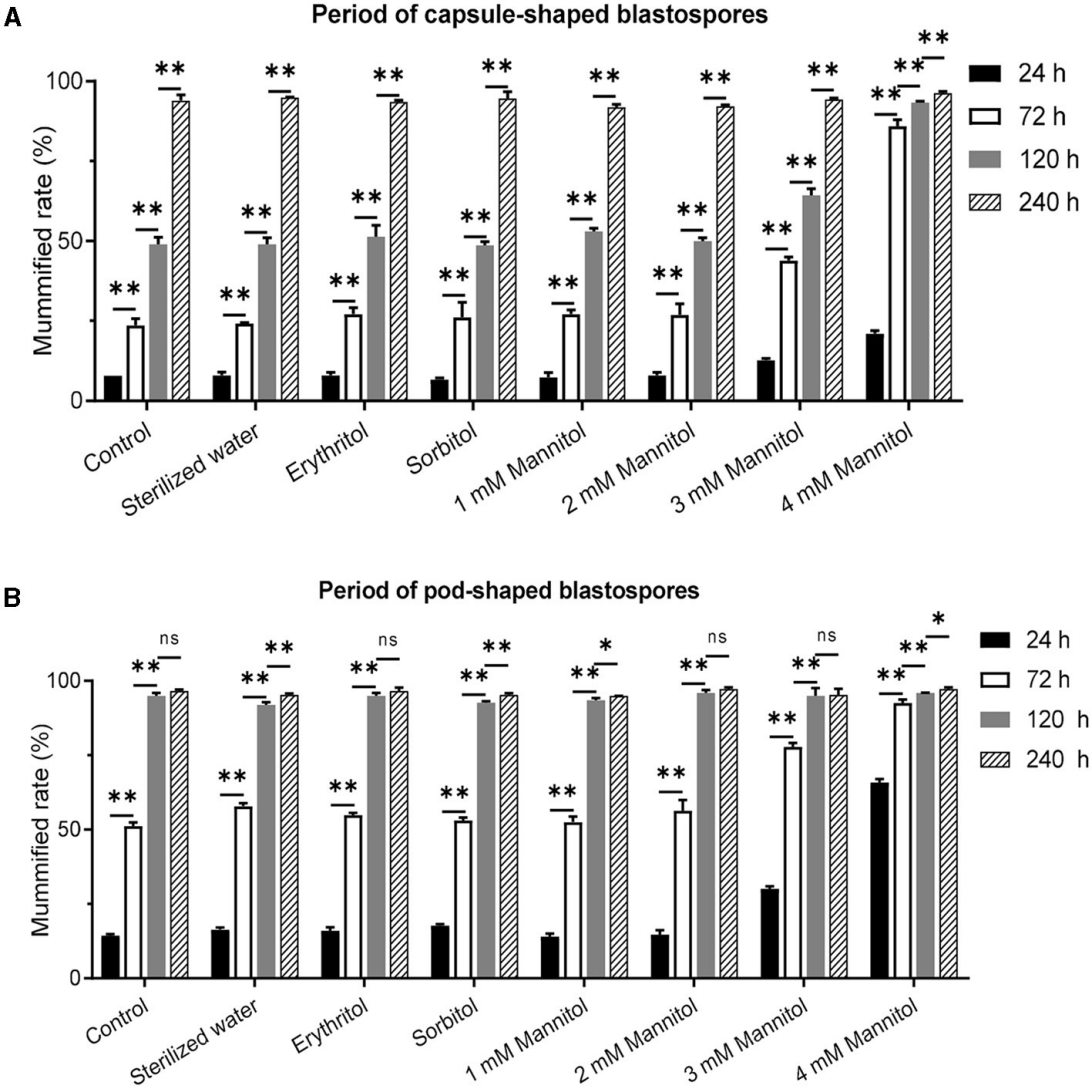
Effects of mannitol injection before and after mummification behavior of infected *T. xiaojinensis* with *O. sinensis* in specific development stage. **(A)** Mummification rate of larvae with capsule-shaped blastospore. **(B)** Mummification rate of larvae with pod-shaped blastospore. Compared with previous stage, data are mean ± SEM. *p < 0.05, **p < 0.01, *N* = 100.

To further validate the effect of mannitol injection on mummification behavior, we microscopically censored and examined infected 8th instar and constant fusiform blastospore in the hemocoel, and the larvae with capsule-shaped blastospores and pod-shaped blastospores were further tested with mannitol injection. The results showed that compared to the control group, the most significant difference in the mummification rate of infected larvae was observed in capsule-shaped blastospores at 72 h after injection of 4 mM mannitol, which was equal at 240 h (Figure 4A). Compared to the natural mummification of the control group, the mummification rate of the infected larvae with capsule-shaped blastospores injected with 4 mM mannitol injection was significantly increased, reaching more than 80%. On the other hand, the most significant difference in the mummification rate was observed at 24 h after injection of 4 mM mannitol in infected larvae with pod-shaped blastospores compared to the control group, and the mummification rate was equal at 120 h (Figure 4B). In conclusion, the mummification rate of larvae increased after mannitol injection, suggesting that mannitol may mediate the mummification of infected larvae.

## Discussion

Chinese cordyceps, a traditional Chinese medicinal herb with high medicinal value in China, is an edible fungus developed by *O. sinensis* parasitizing *T. xiaojinensis* larvae. Insects infected with entomopathogens is known to result in mummification of the insect, which alter the behavior and morphology of the host insect and provide the conditions necessary for the entomopathogen to grow and develop (de Bekker et al., 2021; Elya et al., 2023). For example, summit behavior of zombie ants and fruit flies are influenced by parasitic pathogens, and the hosts climb upward away from food to higher elevations where humidity, temperature, and light are needed before they are killed by the fungus (Andersen et al., 2009; Hughes et al., 2011). In our study of the formation of Chinese cordyceps, *O. sinensis* manipulates *T. xiaojinensis* larvae to exhibit similar behavior. When growing to about 25 days, the *T. xiaojinensis* larvae infected with *O. sinensis* began to crawl slowly, moved away and stopped feeding, and then reached 1–3 cm from the soil surface, with upward-facing head and initial mummified body. The head and mouthparts then stopped moving and the larvae died completely, a phenomenon defined as mummification behavior, and the dead infected larvae are called mummified cadavers. After mummification, the fruit body grows out of the soil to form Chinese cordyceps eventually. Furthermore, we simulated the growth environment of Chinese cordyceps to study the mummification behavior, and found that the infected larvae would lose the ability to crawl and the bodies are mummified, but the heads could still be movable, which is a typical feature of mummification behavior. Meanwhile, the development of the infected larvae and the *O. sinensis* in the body of the mummification larvae before and after mummification behavior were systematically observed, and it was found that the mummification behavior was accompanied by the development of the *O. sinensis* and there was a stable regularity, such as the emergence of the pseudohyphae indicating the occurrence of the mummification behavior. Therefore, the mummified morphology of larvae and the morphology of *O. sinensis* can be used as effective qualitative and quantitative indicators for the study of mummification behavior, and can provide references for future research on insect behavior.

Parasites were driven to alter host behaviors in order to improve their transmission, including spawning, mating, sex pheromones, defensive, and peak behavior (de Bekker et al., 2015). Studies have shown that fungi could alter host behavior by chemical signals, either by releasing chemical effector to modify host physiology and neurobiology or by reducing host nutritional reserves. For example, the smell of spores from the *Metaria* fungus can cause bees, ants, and termites to engage in hygienic behaviors including mutual grooming (Ugelvig and Cremer, 2007; Yanagawa and Shimizu, 2007). Transcriptome analysis of two different *Ophiocordycepsant* interactions suggested that a putative enterotoxin is highly expressed in manipulated summiting and biting behavior (Cooley et al., 2018). The cicadas required specialized symbiotic microorganisms to synthesize essential amino acids to ensure the cicada can use plant sap to live and complete its reproductive behavior (Matsuura et al., 2018). Similarly, we suspect that the emergence of pseudohypha may secrete unique chemical substances to regulate the mummification behavior of ghost moth larvae. Metabolomics has been widely used to reveal the mechanism of parasitic behaviors induced by fungi infection (Barkal et al., 2016; Hautbergue et al., 2018). Pathogenic fungi support their own growth by transferring nutrients from the host, while the host struggles to maintain the stability of the internal environment and cope with the resulting waste, toxins, and tissue damage. Analysis of urine and plasma from *Plasmodium berghei* infected mice revealed disturbances in the gut microbiota and increased levels of pipecolic acid, phenylacetylglycine, and dimethylamine in host urine, and decreased concentrations of taurine (Li et al., 2008). Further, when *Camponotus floridanus* ants were infected by *Ophiocordyceps camponoti-floridani* and exhibited a characteristic summiting phenotype, metabolomic evidence pointed to dysregulation of neurotransmitter levels and neuronal signaling, reporting a metabolic relationship with primary metabolism, detoxification, and anti-stress protectants (Will et al., 2023). The study compared the metabolic differences of the *T. xiaojinensis* larvae before and after mummification behavior by non-targeted and targeted metabolomics methods. We found that changes in glucose and alditol metabolic pathways accompanied the onset of mummification behavior, as well as the appearance of mannitol metabolites. Furthermore, the expression levels of mannitol synthesis-related genes FK, GPI, HK, MPD were significantly up-regulated during mummification, further demonstrating that mannitol plays a key role in mummification behavior. The present study further revealed that the addition of 4 mM mannitol to hemocoel can improve the mummification rate of larvae, indicating that mannitol plays an important role in mummification behavior of infected *T. xiaojinensis* larvae.

Researches have also shown that mannitol plays the role of reactive oxygen species scavengers in the pathogenesis of various plants and animal pathogens, changing conidia to chlamydospore-like structures, thereby enhancing the tolerance of *Gibberella zeae* to heat, drought, and UV light (Solomon et al., 2007; Son et al., 2012; Patel and Williamson, 2016). Besides, mannitol was found to act as a regulator in sexual development and conidial resistance of *Aspergillus fischeri* (Wyatt et al., 2014). MPD is required for mannitol production in vegetative cells, involving in mycelial branching, conidial heat resistance and sexual development in *Aspergillus nidulans* (Lim et al., 2021). Therefore, mannitol may enhance the toxicity of *O. sinensis* by promoting the development of pseudohypha, which would interfere with the interaction between *O. sinensis* and *T. xiaojinensis* larve and cause the host die. Mannitol has been found to play a regulatory role in *Alternaria alternata* development and hyphal stress resistance (Velez et al., 2007). Mannitol accumulates in fungi and acts as a key element in host-pathogen interactions (Meena et al., 2015). Thus, mannitol may enhance the virulence of *O. sinensis*, acting on the death of *T. xiaojinensis* larvae. There are many factors other than mannitol involved in the larvae mummification. For example, N-acetylglucosamine and insect hormone 20-hydroxyecdysone can significantly stimulate hyphal formation in fungi (Liu et al., 2020). When the infected larvae became mummified, *O. sinensis* and *Pseudomonas spp*. became dominant microorganisms, suggesting their possible involvement in larval mummification (Wu et al., 2020). Regrettably, these reports do not provide further validation of the molecular mechanisms or behavioral mediation and regulation of metabolites associated with mummification.

Many treatments to modulate insect behavior are widely used, such as silencing of neurons, gene knockouts, enzyme inhibitor injections, receptor agonists and sterile insects and antibiotics. Knockout of the olfactory receptor gene in the Manduca sexta resulted in changes in foraging behavior, while egg laying behavior remained unchanged (Ye et al., 2017). Injecting tyrosine hydroxylase inhibitors into infected silkworms reduced dopamine levels in brains and successfully blocked virus-induced locomotor behavior (Li et al., 2023). However, this study had some limitations due to the lack of mature confirmed knockout technology for *O. sinensis*, no validation study was conducted with fungus that knocked out mannitol. Secondly, mannitol was the main metabolite studied in this work, it is yet unclear if other metabolites with notable variations have an impact on larval mummification behavior. Furthermore, environmental factors, such as temperature, humidity, and nutritional status of the host, may affect host-parasite interactions. These factors have not been fully considered in this study and further experimental exploration is needed in the future. Even so, our study still provides a useful experimental and data base which can help to elucidate the key role of mannitol in mediating the mummification behavior of *O. sinensis*-infected larvae.

## Conclusion

Overall, metabolomics and molecular biology experiments in this study revealed that mannitol in the hemocoel was a critical regulator of the mummification behavior of *T. xiaojinensis*. Moreover, the injection of 4 mM mannitol into the hemocoel of *T. xiaojinensis* larvae with capsule-shaped blastospore could increase the mummification rate of larvae. This study highlights the key role of mannitol in mediating *O. sinensis*-infected larvae, and provides a possibility for further research on the mechanism of interaction between pathogenic fungi and host insects.

## Data Availability

The original contributions presented in the study are included in the article/Supplementary material, further inquiries can be directed to the corresponding author.
