# Supplementary material for: Mannitol mediates the mummification behavior of Thitarodes xiaojinensis larvae infected with Ophiocordyceps sinensis
Source: Front Microbiol. 2024 Aug 19;15:1411645. doi: 10.3389/fmicb.2024.1411645 (PMC11368059; doi:10.3389/fmicb.2024.1411645)
Supplement: Supplementary file 1 [file Table_1.DOCX]

**Supplemental Material**


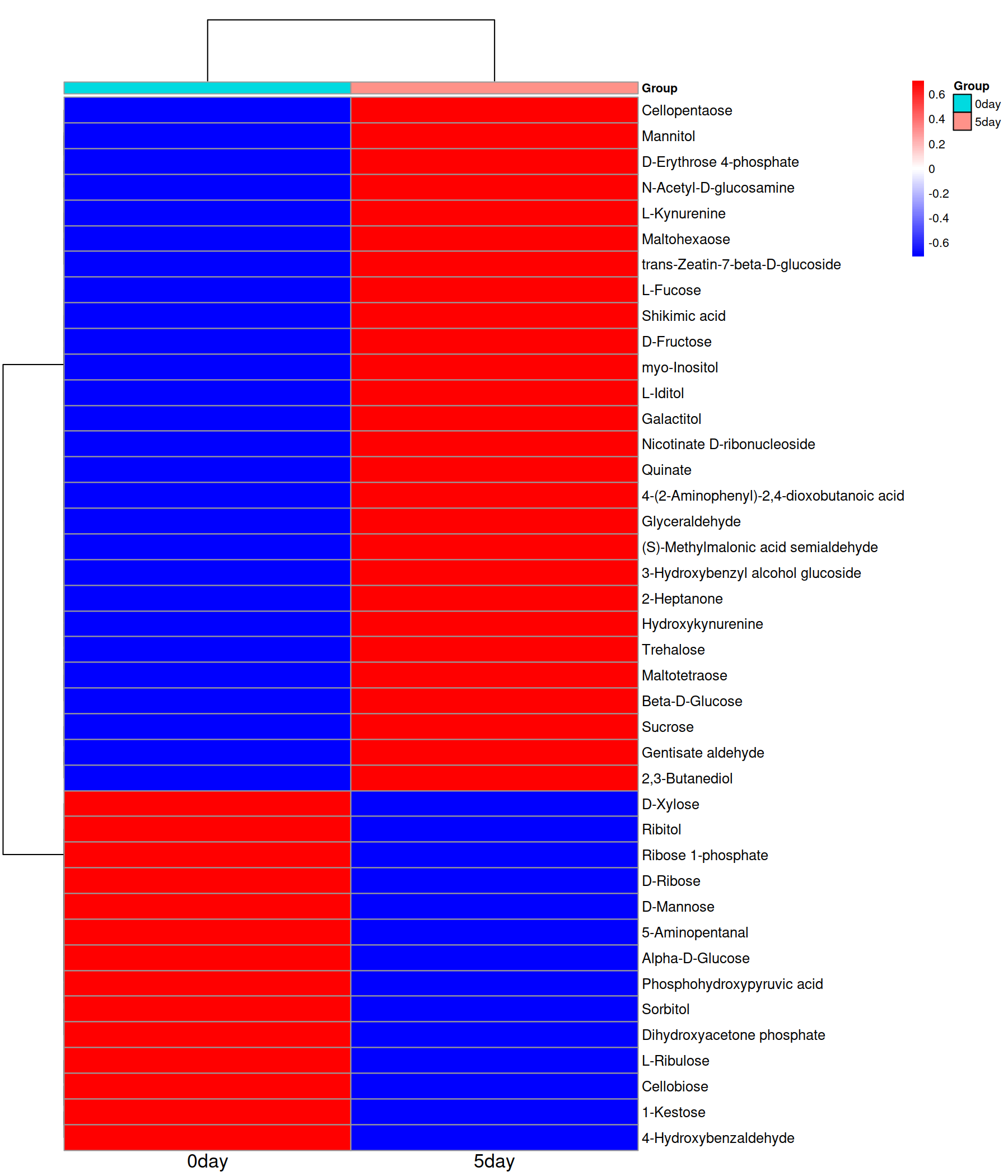


**Supplementary Figure S1. Heatmap analysis.**


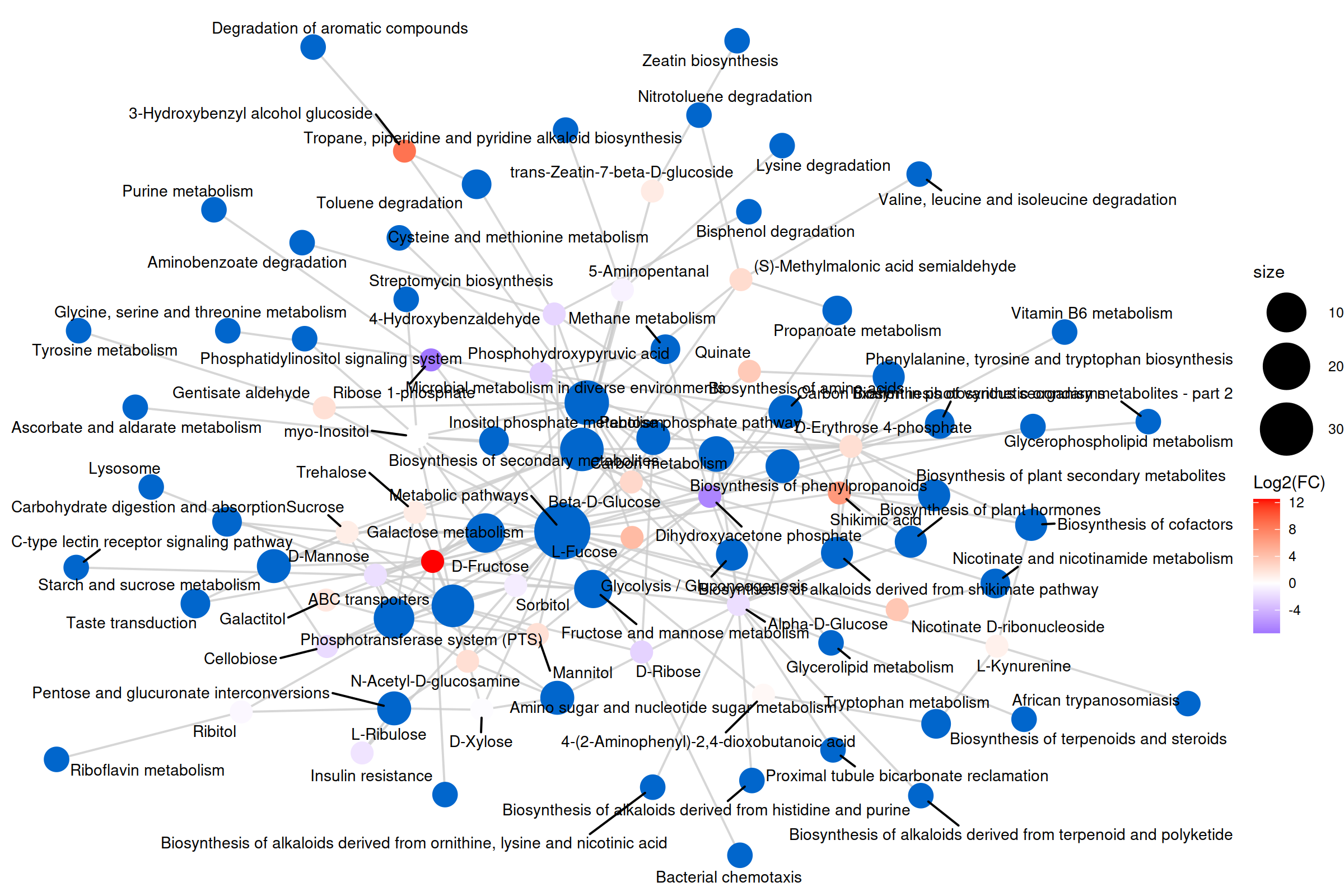


**Supplementary Figure S2. Network result of KEGG analysis.**


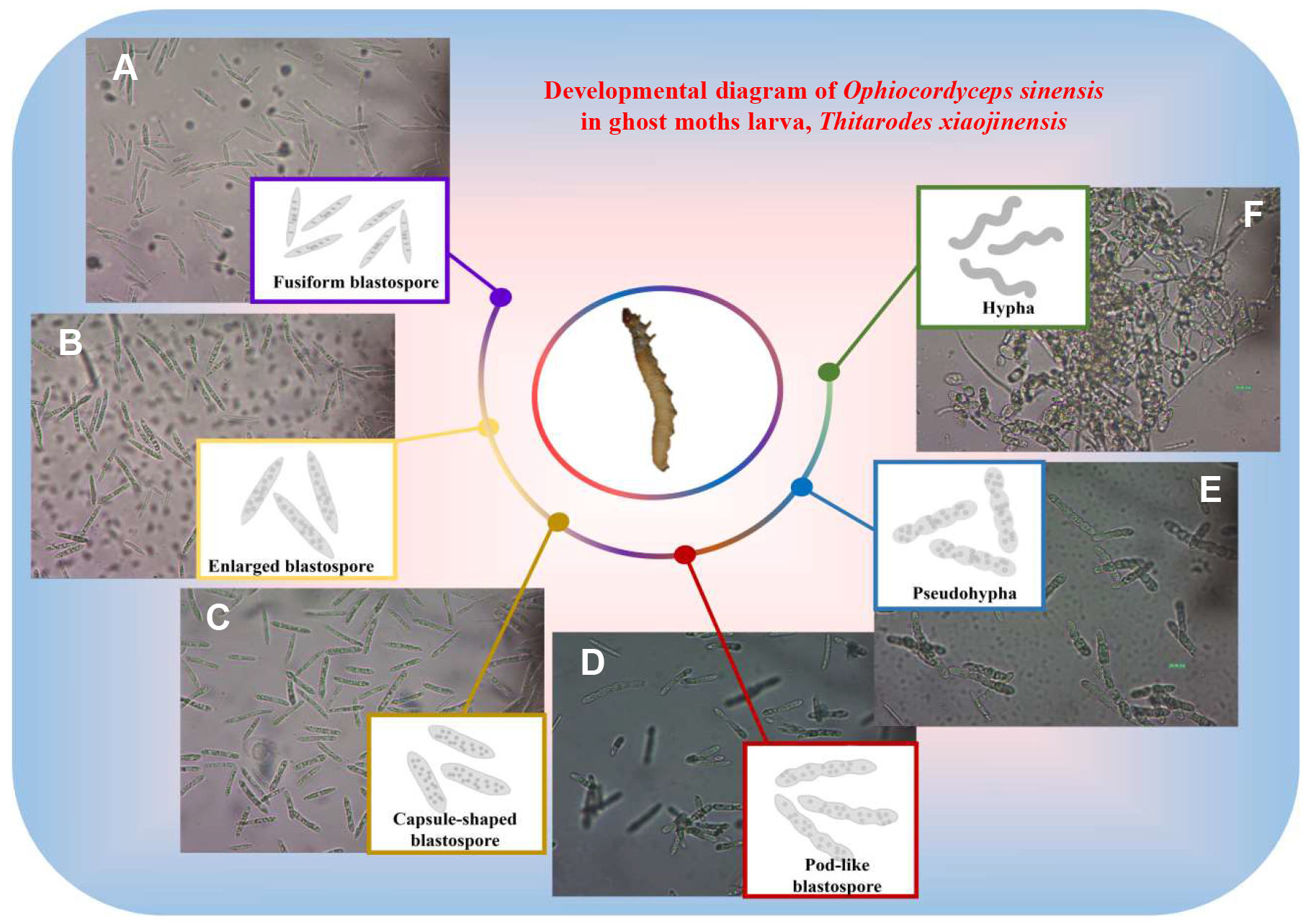


**Supplementary Figure S3. The development process of *O. sinensis*.**

**Supplementary Table S1. Primer sequences.**

| **Gene** | **Gene ID** | **Sequence** | **Length** |
| --- | --- | --- | --- |
| FK -F | KAF4512915.1 | 5′-TTCTTCCCAAACGCCTCTCC-3′ | 103 bp |
| FK -R |  | 5′-CCTGGCCGTTTTCATGGTTG-3′ |  |
| GPI -F | KAF4506005.1 | 5′-GACCAACACTTCCGCAACAC-3′ | 140 bp |
| GPI -R |  | 5′- GCCGGGAAGCGATGAAGATA-3′ |  |
| HK -F | KAF4512602.1 | 5′-TATCCCCATGAACCCGACCT-3′ | 189 bp |
| HK -R |  | 5′- TCGCTTTTGCCCGTTTTGAG-3′ |  |
| MPD -F | KAF4506043.1 | 5′-AGATGTGCTTCCGCTTCCAA-3′ | 88 bp |
| MPD -R |  | 5′-ATGTCCLCGGGTTGGTTGTC-3′ |  |
